# Uracil-DNA Glycosylase Is Involved in DNA Demethylation and Required for Embryonic Development in the Zebrafish Embryo*[Fn FN2]

**DOI:** 10.1074/jbc.M114.561019

**Published:** 2014-04-16

**Authors:** Di Wu, Luxi Chen, Qingrui Sun, Xiaotong Wu, Shunji Jia, Anming Meng

**Affiliations:** From the State-Key Laboratory of Biomembrane and Membrane Engineering, Tsinghua-Peking Center for Life Sciences, School of Life Sciences, Tsinghua University, Beijing 100084, China

**Keywords:** Base Excision Repair (BER), Zebrafish, DNA Demethylation, DNA Transcription, Embryo, DNA Glycosylase, Zygotic Genome Activation, Nuclear Reprogramming

## Abstract

Uracil-DNA glycosylase (Ung) is a component of the base excision repair process and has the ability to remove uracil from U:G mispairs in DNA. However, its implications in development of vertebrate embryos are poorly understood. In this study, we found that zebrafish uracil-DNA glycosylase a (Unga) is maternally expressed at high levels and accumulated in nuclei during cleavage and blastulation periods. Knockdown of *unga* in zebrafish embryos causes an increase of the global DNA methylation level concomitantly with a reduction of overall transcriptional activity in the nucleus, ultimately resulting in embryonic lethality during segmentation period. Conversely, *unga* overexpression is sufficient to reduce the global DNA methylation level, to increase H3K4me3 and H3K27me3 marks, and to activate genome transcription. Furthermore, overexpression of *unga*(*D132A*) mRNA, encoding a mutant Unga without DNA glycosylase activity, does not affect global DNA methylation level, indicating that its involvement in DNA demethylation is dependent on its glycosylase activity. These results together suggest that Unga is implicated in postfertilization genomic DNA demethylation, zygotic gene transcription, and normal embryonic development in zebrafish.

## Teaser

**Background:** Ung implication in DNA demethylation and embryonic development is poorly understood.

**Results:** *unga* knockdown in the zebrafish embryo increases global DNA methylation level, inhibits transcription, and causes embryonic lethality whereas its overexpression produces opposite effects on DNA methylation and transcription.

**Conclusion:** Unga is involved in postfertilization DNA demethylation and transcription.

**Significance:** The findings shed new light on Ung function in DNA demethylation and embryonic development.

## Introduction

The genome of animal embryos at fertilization is transcriptionally inactive and needs to be reprogrammed before the activation of genome transcription and the specification of cell lineages. The reprogramming of the embryonic genome generally involves DNA demethylation, at least in part, which erases 5-methylcytosines (5mC)^2^ inherited from male and female gametes and allows the genome permissive for remodifications (1, 2). In mammals, the paternal genome is rapidly demethylated immediately after fertilization, and the global DNA methylation (GDM) level of the zygotic genome continues decreasing until the morula stage, which is followed by lineage-specific *de novo* methylation (3–7). During *Xenopus* embryogenesis, the GDM level of the genome declines progressively during the cleavage stages and reaches the lowest at the midblastula transition (MBT) (8). It is presumed that the postfertilization demethylation of the zygotic genome in vertebrate embryos is a necessary step for further epigenetic modifications and lineage-specific gene transcription/repression.

In zebrafish embryos, it remains controversial whether parental genomes undergo DNA demethylation immediately after fertilization. Mhanni and Mcgowan disclosed, based on methylation-sensitive restriction enzyme digestion of genomic DNA, that the GDM level of the zygotic genome declines after fertilization and starts to bounce back at blastula stages (9), which was confirmed by immunohistochemistry with an anti-5mC antibody (10). Recently, DNA methylation profiling by whole genome shotgun bisulfite sequencing also detected a moderate decrease of the GDM level during early cleavage stages in zebrafish embryos (11). However, Jiang *et al.* reported that postfertilization demethylation of parental genomes may not occur, and that, upon zygotic genome activation, many loci in maternal chromosomes of embryos are remethylated to the state observed in the sperm (12).

One central question is how the parental genomes in an embryo are demethylated to acquire embryonic totipotency and to activate genome transcription. In mouse zygotes, the dioxygenase TET3 (Ten-Eleven Translocation 3) acts to demethylate the paternal genome by converting 5mC to 5-hydroxymethylcytosine, 5-formylcytosine, and 5-carboxylcytosine before the first mitosis (13–16). However, it seems that dioxygenase-catalyzed DNA demethylation may not function in the zebrafish zygote because 5-hydroxymethylcytosine is rarely detected in early zebrafish embryos (11, 12).

In this study, we identified uracil-DNA glycosylase a (Unga) as a maternally expressed DNA glycosylase. Ung is a member of uracil-DNA glycosylase (UDG) family and participates in base excision repair process by removing uracil from U:G and U:A mispairs in DNA (17). We demonstrate that *unga* is implicated in postfertilization DNA demethylation and zygotic gene transcription activation and is required for normal embryonic development.

## EXPERIMENTAL PROCEDURES

### 

#### 

##### Zebrafish Strain and Microinjection

Tuebingen strain was used in this study with ethical approval from the Animal Care and Use Committee of Tsinghua University. For producing homogeneous embryos, *in vitro* fertilization was performed, and a fraction of embryos was then fixed at desired time points.

When needed, mRNAs, morpholinos, BrUTP, and BrdUTP were individually or in combination microinjected into the yolk or cytoplasm of one-cell stage embryos and collected for analysis at later stages. The dose of BrUTP and BrdUTP was 5 pmol and 1 pmol per embryo, respectively. The mRNAs were injected at a dose of 500 pg per embryo, and morpholinos were injected at a dose of 10 ng per embryo unless otherwise stated. For mRNA synthesis, the coding sequence of zebrafish *unga* (ENSDARG00000042527) was fused to the coding sequence of *mcherry*, which were together subcloned into *pXT7* vector. The mutant form of *unga*, *unga*(*D132A*), was modified from other *unga*-containing vectors. Information on other constructs is described in the other sections. The sequences of unga-MO and cMO are 5′-GCTTTTCTGTCCGATCATTTCCACA-3′ and 5′-GGTTTTGTGTGCCATGATTTCCACA-3′ (mutated bases are underlined), respectively. Embryos were incubated in Holtfreter's solution at 28.5 °C and staged according to Kimmel *et al.* (18).

##### Whole Mount in Situ Hybridization

The antisense RNA probe was *in vitro* synthesized in the presence of digoxigenin-labeled UTP. Whole mount *in situ* hybridization was performed using the commonly used protocol.

##### Immunostaining

Zebrafish embryos at desired stages were fixed by 4% polyformaldehyde for 1 day at 4 °C, dechorionated manually, and dehydrated with methanol. After being stored at −20 °C for 1 h, embryos were rehydrated with 2% PTX (2% Triton X-100 in PBS) and treated in 2 m HCl for 1 h at room temperature followed by neutralizing in 100 mm Tris-HCl (pH 8.5) for 15 min. Embryos were washed with 2% PTX three times, 5 min each, incubated in the block solution (1% BSA, 10% goat serum, 0.3 m glycine in 2% PTX) for 1 h, and transferred to the block solution containing a primary antibody for incubation overnight at 4 °C, followed by washing with 2% PTX six times, 5 min for the first two times and 30 min for the last four times, at room temperature. Next, embryos were incubated in fluorescence-conjugated secondary antibody overnight at 4 °C. After being washed with 2% PTX six times, embryos were mounted and observed by confocal microscopy. Confocal laser scanning was done by the Zeiss LSM710-3 channel system and manipulated by ZEN software. Staining intensity measurement was done by ImageJ software. The following primary antibodies were used: mouse anti-5mC (Abcam ab10805, 1:500), mouse anti-5mC (Abcam ab51552, 1:100), rabbit anti-H3 (Abcam ab1791, 1:500), mouse anti-H3 (EASYBIO BE3015, 1:200), mouse anti-BrdUTP (Santa Cruz sc-32323, 1:100), anti-ssDNA (IBL JP18731, 1:100), rabbit anti-Ung (GeneTex GTX103236, 1:200), rabbit anti-H3K4me3 (Abcam ab8580, 1:100), and rabbit anti-H3K27me3 (Millipore 07-449, 1:100). The secondary antibodies were 488-nm conjugated anti-mouse, 543-nm conjugated anti-mouse, 488-nm conjugated anti-rabbit, and 543-nm conjugated anti-rabbit secondary antibodies (Jackson ImmunoResearch diluted 1:200 for use).

##### DNA Dot Blotting

Genomic DNA was extracted from 256-cell stage embryos using an Animal DNA Kit (DP324, TIANGEN Biotech Co.). All of the purified DNA samples were diluted to a concentration of 50 ng/μl. The diluted samples were denatured at 95 °C for 10 min, quenched on ice and then spotted individually in a desired volume onto a piece of BioTrace^TM^ NT nitrocellulose membrane (Pall Co., T126211), followed by thermal cross-linking at 80 °C for 2 h. The membrane was incubated with the block solution (TBS with 0.5% Tween 20, 5% BSA) for 1 h and then in the block solution containing anti-5mC primary antibody (Abcam ab10805, 1:1000) overnight at 4 °C. The membrane was washed with TBS, 0.5% Tween 20 three times, 10 min each, and then incubated in HRP-conjugated anti-mouse secondary antibody overnight at 4 °C or 1 h at room temperature. Following wash with TBS, 0.5% Tween 20 three times 10 min each, the membrane was incubated in ECL substrate solution for 1 min and then exposed to a film for 5 min.

##### Western Blotting and Cytoplasmic/Nuclear Fractionation Using Embryonic Lysates

Embryos were dechorionated at a desired stage by Pronase treatment and deyolked by pipetting with a 200-μl tip. Embryonic cells were collected after centrifugation at 1000 rpm for 3 min and then lysed in TNE buffer (100 μl for 200 embryos). The lysate was centrifuged at 12,000 rpm for 10 min, and the supernatant was collected. Following addition of SDS loading buffer, the sample was denatured at 95 °C for 10 min, and an aliquot (equivalent to 40 embryos) was loaded onto an SDS-polyacrylamide gel. Cytoplasmic and nuclear proteins were fractionated using the Nuclear and Cytoplasmic Extraction Kit (CWBIO CW0199B) according to the manufacturer's instruction. The used antibodies were: anti-H3 (Abcam ab1791, 1:5000), anti-α tubulin (Sigma T5168, 1:1000), anti-H3 (EASYBIO BE3015, 1:10,000), anti-Ung (GeneTex GTX103236, 1:2000), and anti-actin (Santa Cruz Biotechnology I-19, 1:1000) primary antibodies; HRP-conjugated anti-mouse, HRP-conjugated anti-rabbit, and HRP-conjugated anti-goat secondary antibodies (Jackson ImmunoResearch, 1:5000).

##### Transcriptome Analysis

Total RNA was isolated from zebrafish embryos at the desired stages using the RNeasy Mini Kit (Qiagen 74104). The RNAs were sequenced by BGI Tech using Illumina HiSeq^TM^ 2000 Sequencing System. Sequencing quality evaluation, gene expression annotation, and screening of differentially expressed genes were performed by BGI Tech. Then we did more analysis based on results of DEG (differentially expressed gene) screening.

Genes detected by RNA seq in early wild-type zebrafish embryos were categorized into three classes: Z, M^l^Z^h^, and M^h^Z^l^. For a Z gene, its transcript read was <2/million (two in a million of total transcripts) in one-cell stage embryos likely due to an absence of its maternal transcripts and got higher in 512-cell stage embryos presumably due to its transcription after the zygotic genome activation; the transcript read of a M^l^Z^h^ gene was >2/million in one-cell stage embryos and became further higher in 512-cell stage embryos presumably due to its transcription after the zygotic genome activation; the transcript read of a M^h^Z^l^ gene was more than 2/million in one-cell stage embryos and became lower in 512-cell stage embryos presumably due to degradation of its maternal transcripts and silence of zygotic transcription.

##### DNA Glycosylase Activity Assay

Sequence coding for zebrafish Unga or Unga(D132A) was cloned into the expression vector *pGEX-6P-1*. The recombinant plasmid was transformed into *Escherichia coli* BL21 cells. The expression of the recombinant protein was induced by isopropyl 1-thio-β-d-galactopyranoside at 1 mm for 4 h at 20 °C. The recombinant protein was purified using a nickel-nitrilotriacetic acid spin column. *E. coli* UDG was purchased from New England Biolabs (M0280).

For *in vitro* assay of DNA glycosylase activity, 100-ng DNA oligonucleotides and 30–300 ng of recombinant protein were incubated in 10 μl of 20 mm Tris-HCl, 1 mm EDTA, and 1 mm dithiothreitol for 30 min at 37 °C. The reaction was stopped by adding 90 mm NaOH and 10 mm EDTA and heating at 95 °C for 5 min. The product was separated on polyacrylamide gel followed by ethidium bromide staining.

##### Statistical Analyses

Data averaged from multiple samples were expressed as mean ± S.D. Significance of difference between two treatments was analyzed using Student's *t* test. Significance levels are indicated in the corresponding context.

## RESULTS

### 

#### 

##### The Global DNA Methylation Level Dynamically Changes during Zebrafish Early Embryogenesis

**FIGURE 1.**
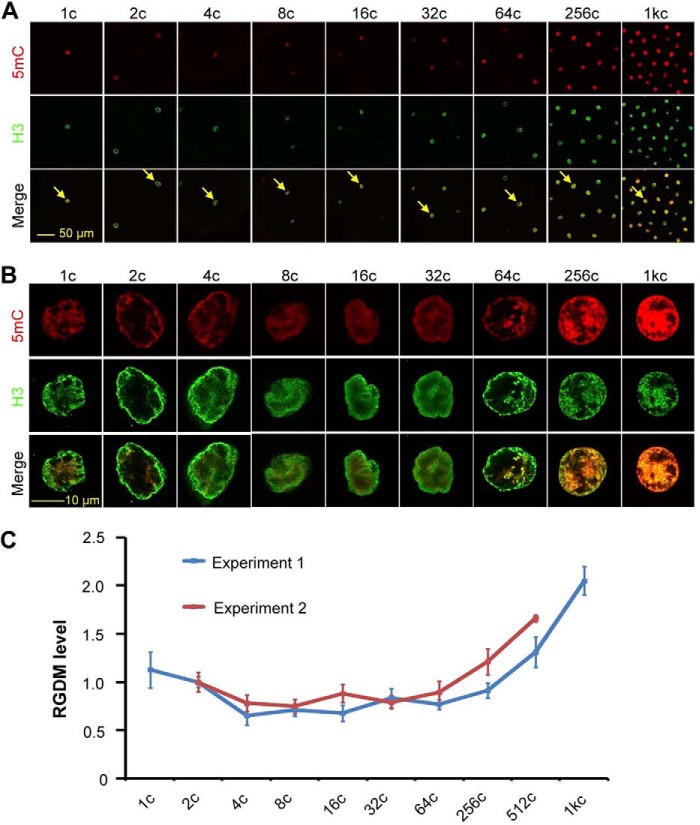
**Dynamic changes of GDM level in early embryos.** *A* and *B*, representative confocal images showing 5mC and H3 signals in embryos at different stages. The *arrow*-indicated nuclei in *A* were imaged at a higher magnification, and corresponding pictures are presented in *B. C*, relative GDM (*RGDM*) level calculated from 5mC and H3 staining signals. The RGDM level is the ratio of 5mC to H3 signal intensity. The data from two independent experiments are shown. For each stage and each experiment, the average was calculated from all nuclei that were clear on the focal plane from at least three embryos. *Error bars* indicate S.D.

Previous papers reported contradictory conclusions about postfertilization DNA methylation pattern during zebrafish early embryogenesis (9–12). We set out to reinvestigate the GDM, by co-immunostaining using anti-5mC and anti-histone 3 (H3) antibodies, during the first 10 cell cycles of zebrafish embryos. The ratio of 5mC/H3 intensities in the nucleus, named relative GDM level, was calculated to allow comparison among embryos at different stages. Results showed that the GDM level tended to decrease gradually from 1-cell (20 min postfertilization) to 8-cell (1.25 h postfertilization (hpf) stages, then maintained roughly unchanged until 32-cell (or 64-cell) stage and thereafter rebounded (Fig. 1). These dynamic changes are similar to those revealed previously by the HpaII/MspI end-labeling assay (9) and by whole genome bisulfite sequencing (see Fig. 1*E* in Ref. 11). Given that the GDM level showed a decrease far earlier prior to MBT, we speculated that maternal factors might contribute to such a demethylation process.

##### unga Transcripts Are Maternally Supplied in Zebrafish Early Embryos

To search for maternal factors possibly related to DNA demethylation, we analyzed RNA profiles of unfertilized eggs and embryos at different stages by RNA seq and found 29 maternal genes whose products have been previously reported to involve DNA demethylation in various species (Table 1). Among them, *unga*, which encodes a uracil-DNA glycosylase superfamily member (19), has the most abundant transcripts in eggs. Its duplicate gene, *ungb*, does not have transcripts in eggs (data not shown). Unexpectedly, transcripts of *tet1-3* and *tdg*, which are known to be involved in active DNA demethylation during early mouse embryogenesis (13–15, 20), were not detected in eggs. Then, our further study had been focused on *unga*.

**TABLE 1:** **Identification of zebrafish genes possibly involving DNA demethylation** mRNAs in squeezed eggs and embryos at different stages were sequenced by RNA seq. The expression levels of zebrafish genes, whose homologs have been previously reported to involve DNA demethylation are shown.

DNA demethylation-related genes		Zebrafish homologs		Normalized transcript number per million at diiferent stages			
Genes	Species*^a^*	Gene ID	Gene names	Egg	1c	512c	1000c
*Tet3*	Mouse (13, 35, 36)	ENSDARG00000062646	*tet3*	0	0	0	0.84
*MBD*	Human (37, 38)	ENSDARG00000025699	*mbd1*	17.21	4.46	31.09	65.56
		ENSDARG00000088407	*mbd1*	7.5	9.75	21.48	8.22
	Mouse (39)	ENSDARG00000004851	*hinfp*	26.93	15.04	22.04	172.71
		ENSDARG00000037405	*zgc:112083*	59.42	74.66	26	74.11
		ENSDARG00000014218	*mecp2*	0	0	0.57	4.36
	Zebrafish (33)	ENSDARG00000061774	*mbd3a*	88.29	239.57	49.17	56.84
		ENSDARG00000042029	*mbd3b*	0.83	0.56	29.11	13.92
*Gadd45*	Zebrafish (33)	ENSDARG00000044676	*gadd45g*	1.11	0.56	0.57	9.73
	*Xenopus* (40)	ENSDARG00000019417	*gadd45g*	0	0	9.33	1.84
	Mouse (41)	ENSDARG00000027744	*gadd45ba*	0	0	85.91	5.87
	Human (41)	ENSDARG00000043581	*gadd45aa*	0	0	2.26	0.67
		ENSDARG00000013576	*gadd45bb*	0.83	0	36.45	34.04
*DNA Glycosylase*	Human (42)	ENSDARG00000086450	*tdg*	2.5	3.34	1.98	21.97
		ENSDARG00000013004	*tdg*	0	0	1.13	0.67
	*Arabidopsis* (43, 44)	ENSDARG00000042527	*unga*	765.2	537.91	85.06	103.96
		ENSDARG00000069729	*mpg*	0	0	1.7	1.51
		ENSDARG00000094922	*ungb*	0	0	0	0.84
	Chick (45, 46)	ENSDARG00000074889	*mutyh*	1.67	23.4	8.48	16.77
		ENSDARG00000087197	*ros1*	0.56	0	0	0.5
*Endonuclease*	Chick (45, 47)	ENSDARG00000045843	*apex1*	272.93	412.28	46.35	35.38
		ENSDARG00000018061	*neil1*	1.39	1.95	42.11	28.17
		ENSDARG00000042881	*nthl1*	19.16	12.54	17.8	19.12
	Mouse (47, 48)	ENSDARG00000020079	*neil3*	123.83	70.2	33.63	80.65
		ENSDARG00000074889	*mutyh*	1.67	23.4	8.48	16.77
		ENSDARG00000063535	*chd4a*	23.04	183.3	85.63	13.41
	Human (48, 49)	ENSDARG00000004274	*zgc:112496*	0	0.56	2.26	3.86
		ENSDARG00000023678	*ercc5*	1.67	0	3.11	2.68
		ENSDARG00000014161	*ercc4*	59.14	51.81	21.76	56.01
*Alkb*	*E. coli* (50, 51)	ENSDARG00000077253	*alkbh6*	0.56	0	6.5	5.2
		ENSDARG00000059856	*alkbh2*	1.39	3.06	0	0.84
		ENSDARG00000045606	*alkbh3*	9.44	8.91	3.67	12.58
		ENSDARG00000071164	*alkbh1*	4.44	13.37	8.48	12.74
		ENSDARG00000037906	*alkbh7*	0	0	9.04	18.95
		ENSDARG00000063003	*alkbh5*	1.11	7.52	61.32	118.38
		ENSDARG00000052247	*alkbh4*	321.24	103.35	0.85	10.06
		ENSDARG00000060256	*alkbh8*	1.11	37.61	38.15	26.49
*Deaminase*	Zebrafish (33)	ENSDARG00000034604	*apobec2b*	0	0	0.85	0
	Mouse (52)	ENSDARG00000036990	*dctd*	25.54	10.31	7.63	26.16
	Human (52–55)	ENSDARG00000036426	*zgc:103586*	2.78	2.23	5.93	3.69

*^a^* Related references are indicated.

**FIGURE 2.**
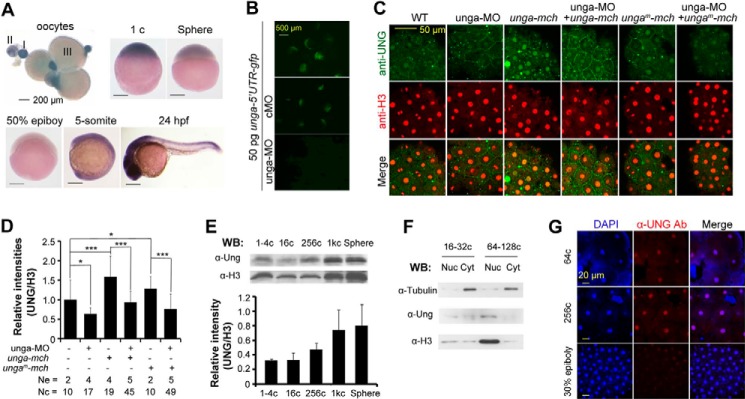
**Temporal expression and subcellular localization of Unga.** *A*, expression of *unga* in eggs and embryos at different stages, detected by whole mount *in situ* hybridization. Egg stages (I–III) and embryonic stages are indicated. *B*, effect of unga-MO on a reporter expression. The reporter construct *unga-5*′-*UTR-gfp* was made by ligating 73 bp of 5′-UTR and immediate downstream 291-bp coding sequence of *unga* cDNA to *gfp* coding region in pEGFP-N3. Embryos were injected with 50 pg of reporter plasmid DNA in combination with 10 ng of unga-MO or the control cMO at the one-cell stage and observed under a fluorescence stereomicroscope during midgastrulation stages. *C* and *D*, anti-human Ung antibody recognized Unga protein in fish embryos. Embryos at the one-cell stage were injected with 10 ng of unga-MO, 500 pg of *unga-mcherry* mRNA, or 500 pg of *unga^m^-mcherry* mRNA alone or in different combinations and harvested at the 1000-cell stage for co-immunostaining using anti-UNG and anti-H3 antibodies (*C*). *unga^m^-mcherry* was identical to *unga-mcherry* except several mutated bases in unga-MO recognizing sequence. The relative immunostaining intensity of Unga, which was normalized to the H3 signal, is shown in the *bar graph* (*D*). *Ne*, number of measured embryos; *Nc*, total number of measured nuclei. Statistical significance: *, *p* < 0.1; **, *p* < 0.05; ***, *p* < 0.01. *Error bars*, S.D. *E*, detection of Unga protein at different developmental stages. Embryos were collected at the indicated stages and lysed for Western blotting using anti-Ung or anti-histone 3 (H3) antibody. The *bottom bar graph* shows the average Unga signal level relative to H3 signal intensity. *F*, detection of subcellular localization of Unga protein. Cell lysates from embryos at different stages were fractionated into nuclear (*Nuc*) and cytoplasmic (*Cyt*) fractions, which were then subjected to Western blotting using anti-Ung, anti-H3, or anti-tubulin antibody. Note that the fractions were not very clean due to fast cell division without G_1_ and G_2_ phases during the cleavage period. *G*, Unga protein located in nuclei in embryos at different stages and detected by immunofluorescence with anti-Ung antibody.

We investigated the spatiotemporal expression pattern of *unga* by whole mount *in situ* hybridization. Transcripts of *unga* were present in immature eggs of different stages and in embryos with uniform distribution during early cleavage period (Fig. 2*A*). Its expression was reduced to an undetectable level at the 50% epiboly stage (5.3 hpf) and resumed after the bud stage. During segmental period, its transcripts were ubiquitously present with enrichment in the neural tube and tailbud. These results imply that *unga* may play distinct roles during cleavage period and segmentation period.

##### Unga Protein Is Located in Nuclei of Blastomeres of Zebrafish Embryos

If Unga functions immediately after fertilization, Unga protein should be present in early embryos. Currently there are no anti-zebrafish Unga antibodies available, prompting us to test the utility of an anti-human UNG antibody in fish. To verify the efficacy of this antibody, we designed the antisense morpholino unga-MO to block the translation of endogenous *unga* mRNA. Injection with unga-MO, but not with the mismatched control MO (cMO), inhibited the expression of the reporter *unga-5*′-*UTR-gfp* (Fig. 2*B*), demonstrating an effective blockage. We found that immunostaining signal using anti-human UNG antibody was enhanced by overexpression of *unga-mcherry* mRNA and decreased by *unga* knockdown and showed a mutual rescuing effect if both mRNA and MO were injected (Fig. 2, *C* and *D*), suggesting that this antibody reacts with endogenous Unga protein in zebrafish embryos. By Western blotting, we found that Unga protein was detectable in embryos from the one-cell stage onward (Fig. 2*E*). Analysis of nuclear and cytoplasmic fractions indicated that Unga protein was predominantly present in nuclei (Fig. 2*F*). Immunofluorescence assay could detect weak Unga signal in nuclei of 4-cell stage embryos (data not shown) and stronger signals in nuclei in embryos at later stages (Fig. 2*G*). Thus, Unga is similar to human UNG2 that is localized in the nucleus (21), suggesting that Unga functions in the nucleus.

##### Unga Possesses Uracil Excision Activity

**FIGURE 3.**
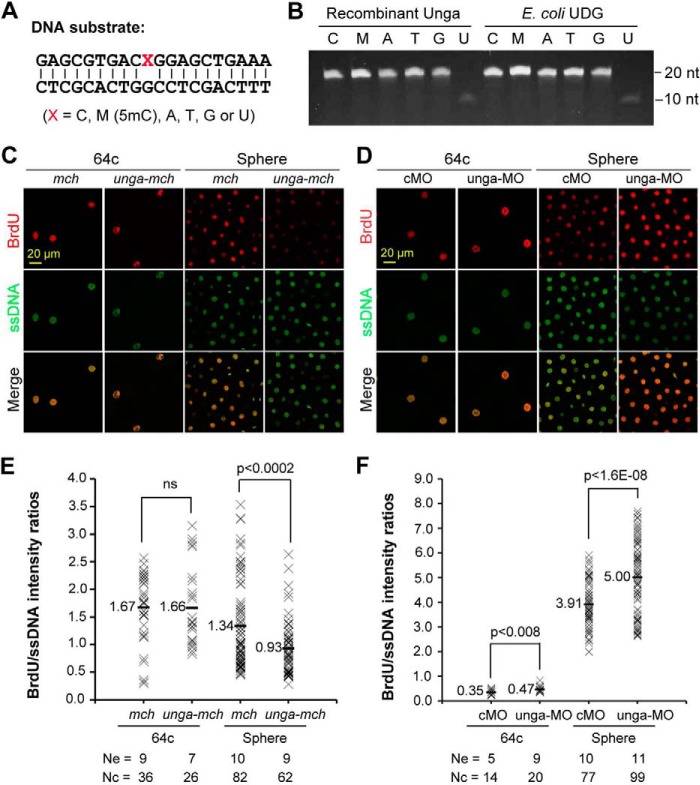
**Unga functions to excise uracil bases from DNA.** *A* and *B*, *in vitro* assay of uracil excision activity of Unga. The sequences of 20-bp double-stranded DNA oligonucleotides are shown in *A*. DNA substrates were incubated for 30 min at 37 °C with 30 ng/μl recombinant Unga or bacterial UDG, then stopped and separated on a polyacrylamide gel and stained (*B*). *C* and *D*, confocal fluorescence of incorporated BrdU. Embryos were co-injected at the one-cell stage with 1 pmol of BrdUTP and 500 pg of *mcherry* (*mch*) or 500 pg of *unga-mcherry* (*unga-mch*) mRNA, or 10 ng of cMO or 10 ng of unga-MO, and fixed at 64-cell and sphere stages for immunostaining with anti-BrdU and anti-ssDNA antibodies. *E* and *F*, relative intensities of BrdU signals. The BrdU incorporation level is reflected by the ratio of BrdU/ssDNA intensities in a nucleus. The ratios are shown in a string of *crosses* for all analyzed nuclei of the same treatment with an average indicated. *Ne*, number of observed embryos; *Nc*, total number of analyzed nuclei. *ns*, not statistically significant (*p* > 0.1).

Bacterial and mammalian UDG/Ung proteins have activity to excise uracil from DNA (17, 22, 23). We tested whether zebrafish Unga possesses similar activity. We found that, like *E. coli* UDG (22), recombinant Unga efficiently released uracil from mispairing U:G in double-stranded oligonucleotides in *in vitro* assays (Fig. 3, *A* and *B*). However, we did not detect excision of other mispairing bases such as T:G, A:G, G:G, C:G, or 5mC:G.

We further investigated the uracil excision activity of Unga in zebrafish embryos through dUTP incorporation assay. Embryos were injected at the one-cell stage with BrdUTP in combination with *mcherry* or *ung-mcherry* mRNA, cMO, or unga-MO and fixed at 64-cell and sphere stages for co-immunostaining using anti-BrdU and anti-ssDNA antibodies. The relative BrdUTP incorporation level was estimated as the ratio of BrdU signal intensity to ssDNA signal intensity in nuclei. Results showed that *unga* knockdown enhanced but *unga* overexpression hindered incorporation of dUTPs into newly synthesized DNA in zebrafish embryos (Fig. 3, *C–F*), supporting the notion that Unga can function to remove uracil bases from DNA molecules.

##### unga Knockdown Increases and Overexpression Decreases Global DNA Methylation Level in Zebrafish Pre-MBT Embryos

**FIGURE 4.**
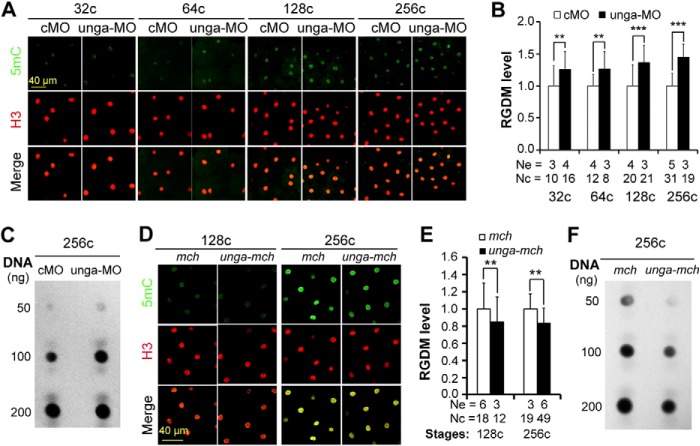
**Effect of *unga* knockdown or overexpression on the global DNA methylation level during early embryogenesis.** *A, B*, *D*, and *E*, immunodetection of 5mC and H3 in nuclei (*A* and *D*) and the 5mC/H3 ratio (RGDM) (*B* and *E*). Embryos were injected at the one-cell stage with 10 ng of cMO, 10 ng of unga-MO, 500 pg of *mcherry* (*mch*) or *unga-mcherry* (*unga-mch*) mRNA and fixed at indicated stages for immunostaining. The RGDM was averaged from multiple nuclei and three to five embryos for each treatment. *Ne*, number of measured embryos; *Nc*, number of measured nuclei. **, *p* < 0.05; ***, *p* < 0.01. *Error bars*, S.D. *C* and *F*, detection of 5mC level by dot blotting using genomic DNA isolated from 256-cell stage embryos and anti-5mC antibody.

A recent paper reported a possible involvement of Ung2 in active DNA demethylation in the mouse zygote (24). We asked whether *unga* was implicated in DNA demethylation during zebrafish early embryogenesis. When *unga* was knocked down by injecting 10 ng of unga-MO, embryos exhibited a significant increase of the relative GDM level as early as the 32-cell stage and at later stages as estimated by immunostaining with anti-5mC antibody (Fig. 4, *A* and *B*). The increase in GDM level in *unga* morphants at the 256-cell stage was also confirmed by dot blotting (Fig. 4*C*). Conversely, overexpression of *unga-mcherry* mRNA in zebrafish embryos resulted in a significant decrease of the relative GDM level, as detected by immunostaining at 128-cell and 256-cell stages (Fig. 4, *D* and *E*) and by dot blotting at the 256-cell stage (Fig. 4*F*). These results together suggest that Unga is involved in engendering and maintaining low levels of DNA methylation in the embryonic genome during pre-MBT period of zebrafish embryos.

##### Unga-mediated DNA Demethylation Relies on DNA Glycosylase Activity

**FIGURE 5.**
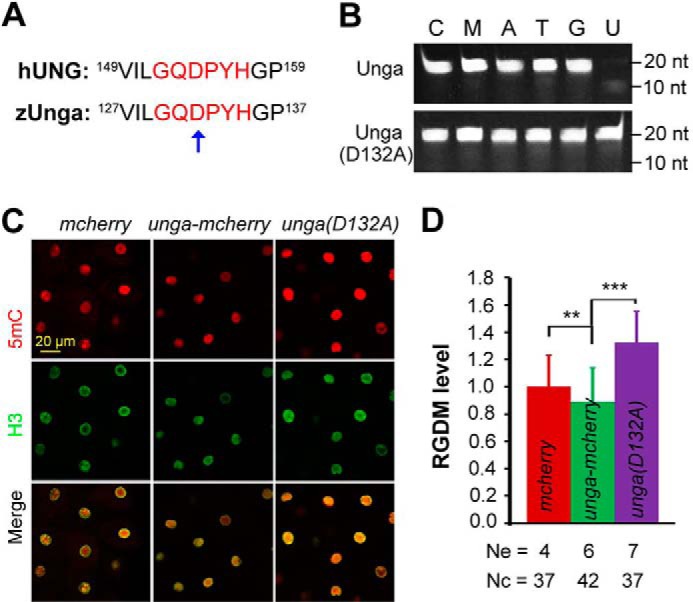
**DNA glycosylase activity of Unga is required for DNA demethylation activity.** *A*, conserved water-activating loop (*red letters*) between human (hUNG2) and zebrafish Ung (zUnga). The mutated residue in Unga(D132A) is indicated by an *arrow. B*, comparison of uracil excision activity of Unga and Unga(D132A). Sequence information for DNA oligonucleotides is the same as shown in Fig. 3*A*. The oligonucleotide was incubated with the recombinant protein (3 ng/μl) for 30 min at 37 °C. *C* and *D*, immunofluorescence of injected embryos at the 256-cell stage using anti-5mC and anti-H3 antibodies (*C*) and the relative global DNA methylation (RGDM) levels shown in *D. Ne*, number of measured embryos; *Nc*, total number of measured nuclei. **, *p* < 0.05; ***, *p* < 0.01. *Error bars*, S.D.

Our next question was whether Unga-mediated DNA demethylation depended on its DNA glycosylase activity. Because the aspartate residue in the conserved water-activating loop motif GQDPY of Ung proteins has been shown to be critical for its catalytic activity (25), we generated a presumably inactive mutant of zebrafish Unga, Unga(D132A), which carried a mutation to alanine at position 132 (Fig. 5*A*). An *in vitro* assay confirmed that Unga(D132A) had much reduced activity to excise uracil bases from double-stranded DNA (Fig. 5*B*). Injection of *unga*(*D132A*) mRNA was unable to reduce the global DNA methylation level at the 256-cell stage; it instead resulted in an increase of the methylation level (Fig. 5, *C* and *D*), which might be due to a dominant negative effect. Nevertheless, we conclude that Unga regulation of DNA methylation is dependent on its glycosylase activity.

##### Unga-mediated DNA Demethylation Facilitates Histone Modifications of Chromatin in Zebrafish Embryos

**FIGURE 6.**
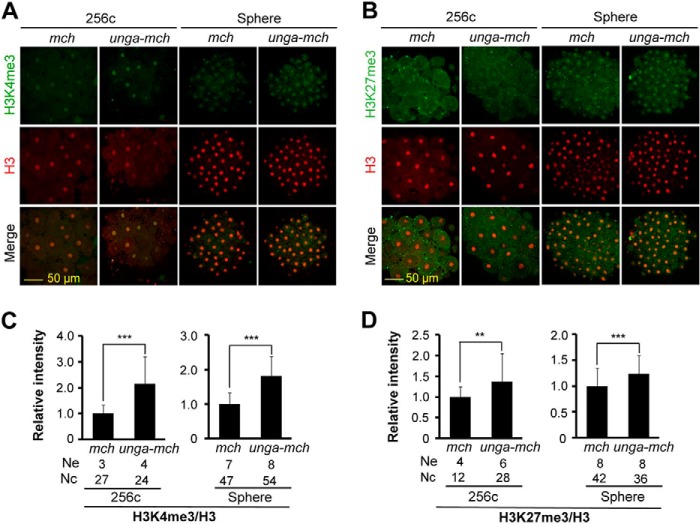
**Histone modifications are facilitated by *unga* overexpression.** *A* and *B*, representative confocal fluorescence images of embryos following co-immunostaining using anti-H3K4me3 (*A*) or anti-H3K27me3 (*B*) together with anti-H3 antibodies. Embryos were injected at the one-cell stage with 500 pg of *mcherry* (*mch*) or *unga-mcherry* (*unga-mch*) mRNA and fixed at 256-cell and sphere stages for immunostaining. *C* and *D*, relative immunostaining intensity of H3K4me3 (*C*) and H3K27me3 (*D*) normalized to H3. *Ne*, number of measured embryos; *Nc*, total number of measured nuclei. **, *p* < 0.05; ***, *p* < 0.01. *Error bars*, S.D.

Methylated histones in zebrafish embryos are observed just before and during MBT, marking chromatin regions for transcription or repression (26, 27). We asked whether Unga-mediated DNA demethylation would poise specific chromatin regions for histone modifications. To this end, we injected embryos at the one-cell stage with 500 pg of *mcherry* or *unga-mcherry* mRNA and examined H3K4me3 and H3K27me3, together with H3, at 256-cell and sphere stages by immunostaining with corresponding antibodies. Compared with *mcherry*-injected embryos, *unga-mcherry*-injected embryos exhibited an increase of the relative intensity of both H3K4me3 and H3K27me3, which was normalized to H3 intensity (Fig. 6, *A–D*). These results indicate that the genome may be unfettered by *unga*-stimulated hypomethylation for histone modifications.

##### unga Knockdown Inhibits Yet Its Overexpression Stimulates Nuclear Transcriptional Activity

**FIGURE 7.**
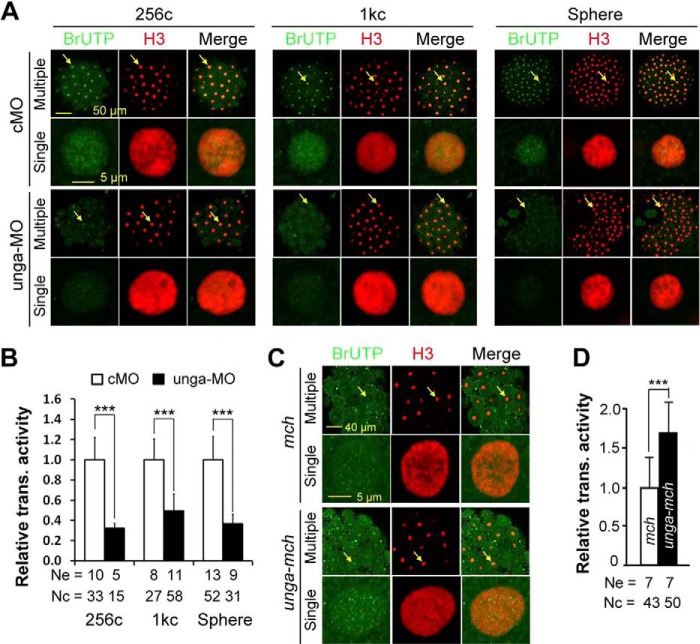
***unga* knockdown or overexpression affects nuclear transcriptional activity.** Embryos at the one-cell stage were injected with 5 pmol of BrUTP in combination with 10 ng of cMO, 10 ng of unga-MO, 500 pg of *mcherry* (*mch*) mRNA or 500 pg of *unga-mcherry* (*unga-mch*) mRNA and fixed at indicated stages for immunostaining with anti-BrUTP and anti-H3 antibodies. The immunostained embryos were imaged by confocal microscopy. The relative transcriptional activity is the ratio of BrUTP to H3 signal intensity in the nucleus. *A* and *B*, representative confocal images (*A*) and the average relative transcriptional activity (*B*) in MO-injected embryos. *C* and *D*, representative confocal images (*C*) and the average relative transcriptional activity (*D*) in mRNA-injected embryos. In image panels, a selected nucleus (indicated by an *arrow*) in the representative embryo is enlarged underneath. *Ne*, number of measured embryos; *Nc*, total number of measured nuclei. ***, *p* < 0.01. *Error bars*, S.D.

Given that *unga* may participate in postfertilization DNA demethylation and regulate histone modifications, we further tested whether its knockdown or overexpression altered global transcriptional activity in the nucleus. We labeled newly synthesized RNAs by injecting 5-BrUTP together with unga-MO or *unga-mcherry* mRNA into one-cell stage embryos and then visualized incorporated BrUTPs by immunostaining embryos at different stages with anti-BrUTP and H3 antibodies. The relative transcriptional activity of the genome was assessed as the ratio of BrUTP immunostaining intensity to H3 intensity in nuclei. Compared with cMO-injected embryos, embryos injected with 10 ng of unga-MO exhibited a significant decrease of the relative transcriptional activity at 256-cell, 1000-cell, and sphere stages (Fig. 7, *A* and *B*). In contrast, *unga-mcherry-*injected embryos at the 256-cell stage exhibited an increase of overall transcriptional activity in nuclei compared with *mcherry*-injected embryos (Fig. 7, *C* and *D*). These results imply that insufficiency of Unga may repress transcriptional activity of the genome due to higher levels of DNA methylation, and that excess Unga may stimulate nuclear transcriptional activity arising from DNA hypomethylation.

##### Zygotic Expression of Many Genes Is Altered with Changes of Unga Levels in Zebrafish Embryos

**FIGURE 8.**
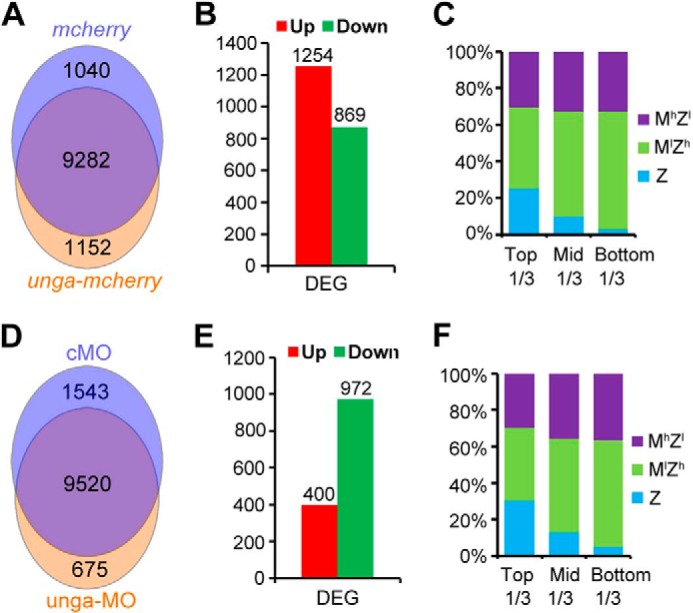
**RNA seq analyses of *unga*-overexpressing and *unga* knockdown embryos.** *A–C*, comparison of transcriptional profile between *mcherry*- and *unga-mcherry*-injected embryos at the 256-cell stage. *A*, gene numbers mapped to the zebrafish genome assembly version Zv9 in different samples. *B*, number of up-regulated or down-regulated genes between two samples (>1.5-fold with false discovery rate <0.005). *C*, the ratios of up- and down-regulated genes in different classes. The DEGs were grouped into three sets (for details, see “Experimental Procedures”) based on degrees of -fold changes from the highest to the lowest: top third, middle third, and bottom third. *D–F*, comparison of transcriptional profile between cMO- and unga-MO-injected embryos at the 1000-cell stage. The DEGs were analyzed in ways similar to those in mRNA-injected embryos.

To further understand the profound impact of Unga-mediated demethylation on zygotic gene transcription, we performed genome-wide mRNA seq analysis for *unga-mcherry*-injected embryos at the 256-cell stage and *unga* morphants at the 1000-cell stage (Fig. 8). For each sample, transcripts of more than 10,000 genes were mapped to the zebrafish genome assembly version Zv9 (Fig. 8, *A* and *D*). Compared with *mcherry*-injected embryos, *unga-mcherry-*injected embryos at the 256-cell stage had 1254 up-regulated and 869 down-regulated genes (>1.5-fold with a false discovery rate <0.005) (Fig. 8*B* and supplemental Tables S2 and S3). Validation of up-regulated genes by RT-PCR analysis revealed that 26 of 33 genes indeed showed a significant increase of expression by *unga-mcherry* overexpression (data not shown).

By comparing expression levels in between one-cell and 512-cell stage embryos based on our other RNA seq data (supplemental Table S1), we categorized all genes into three classes: M^l^Z^h^ (with increasing amount of transcripts from the one-cell stage onward), Z (zygotically transcribed), and M^h^Z^l^ (with decreasing amount of transcripts after the one-cell stage). More than 90% of the DEGs identified in this study could be successfully classified. We found that the majority of the DEGs between *unga-mcherry*- and *mcherry*-overexpressing embryos were Z or M^l^Z^h^ genes and that differently expressed Z genes mostly fell within the top one-third group as ranked by changing fold (Fig. 8*C*), supporting the idea that *unga*-promoted DNA hypomethylation may evoke zygotic transcription. Among the up-regulated genes in *unga-mcherry* embryos, many are related to nucleotide excision repair, base excision repair, or mismatch repair pathways (supplemental Table S2), suggesting that ectopic Unga induces a more active DNA repair system; and many are required for general transcription, which is consistent with enhanced genome transcriptional activity. Interestingly, some up-regulated genes could not function normally before cell lineage specification, *e.g. klfd*, which is normally expressed after the completion of gastrulation and crucial for hematopoiesis (28–30), and *gli3*, which controls neural induction and patterning (31, 32). It is likely that cell lineages could be abnormally specified due to hypomethylation.

Comparison between unga-MO- and cMO-injected embryos at the 1000-cell stage identified 972 down-regulated and 400 up-regulated genes in *unga* morphants (Fig. 8*E* and supplemental Tables S4 and S5). Validation of down-regulated genes by RT-PCR analysis revealed that 30 of 35 genes showed significantly decreased expression levels in *unga* morphants (data not shown). We noted that the majority of differentially expressed genes were also Z or M^l^Z^h^ genes (Fig. 8*F*). These results suggest that many genes required for early embryonic development are repressed for transcription probably due to hypermethylation of the genome. Among the down-regulated genes in *unga* morphants, some are also related to transcription, which accords with a repression of general transcriptional activity in the morphants. Interestingly, knockdown of *unga* led to significant up-regulation of genes related to cell death, *e.g. bax*, *pcdc2*, *pcdc5*, *daxx*, and *dido1*, which might be one of the reasons for embryonic lethality of *unga* morphants (see below).

##### unga Knockdown Causes Embryonic Lethality during Segmentation Period

**FIGURE 9.**
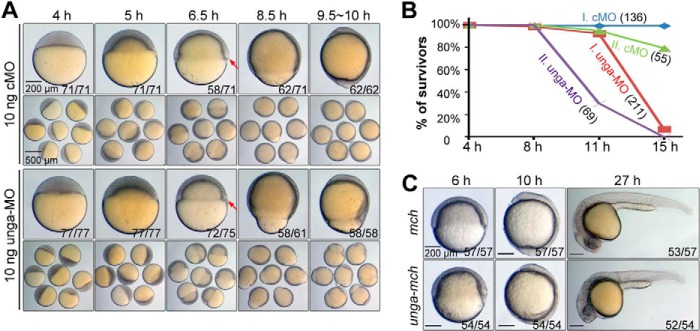
**Effects of *unga* knockdown and overexpression on embryonic development.** *A*, morphology of embryos. Embryos were injected with 10 ng of cMO or unga-MO at the one-cell stage and imaged individually (*top panel*) or in a group (*bottom* p*anel*) at indicated stages. Individual embryos were viewed laterally with dorsal to the right if the dorsal side was distinguishable. The ratio of embryos with representative morphology is indicated in the *right corner*. The embryonic shield is indicated by an *arrow. B*, surviving rates of embryos at different time points of development. Data from two independent experiments (*I* and *II*) are shown with the number of embryos at 4 hpf in *parentheses. C*, morphology of embryos injected with 500 pg of *mcherry* (*mch*) mRNA or *unga-mcherry* (*unga-mch*) mRNA at the indicated stages. The ratio of embryos with representative morphology is indicated.

We observed morphological changes of embryos depleted of *unga* during development (Fig. 9*A*). Like control embryos injected with cMO, embryos injected with 10 ng of unga-MO had no observable defects before 40% epiboly stage (5 hpf) except a slightly retarded epiboly; then, *unga* morphants continued to show a slower epibolic process and had a thinner germ ring with a smaller embryonic shield around the shield stage. A large proportion of morphants started to deform at the onset of segmentation, and none of them could survive beyond 12-somite stage (approximately 15 hpf) (Fig. 9*B*). These results imply that Unga is absolutely required for survival of zebrafish embryos. In contrast, embryos injected with 500 pg of *unga-mcherry* mRNA could develop beyond pharyngula period without severe morphological defects (Fig. 9*C*). It is likely that temporary DNA hypomethylation during early development may not severely disrupt normal developmental programs.

## DISCUSSION

In this study, we uncovered an implication of the base excision repair system in reprogramming the embryonic genome through DNA demethylation during zebrafish early embryogenesis. This function is mediated, at least in part, by Unga, a uracil-DNA glycosylase family member.

Currently, we do not know how Unga participates in DNA demethylation during zebrafish early embryogenesis. Our *in vitro* DNA glycosylase activity assays indicated that recombinant Unga alone is able to excise uracil base from mispairing U:G in double-stranded DNA, but not other bases (including 5mC) pairing with G (Fig. 3*B*). In physiological conditions, uracil bases could result from deamination of cytosine bases and misincorporation of dUMPs. There might be several ways for Ung to take part in DNA demethylation. First, excision of misincorporated uracil bases by Ung may be followed by excision of a long stretch of adjacent nucleotides including 5mC, resulting in a replacement of 5mC with C during subsequent repair process. This mechanism may not be responsible for the genome-wide demethylation because the genome may not contain many misincorporated U bases.

Second, Ung may be involved in deaminase-mediated DNA demethylation because 5mC bases can be converted to thymine/cytosine bases through the AID/Apobec2/Gadd45/Mbd4 system (33, 34), and cytosine bases can be further deaminated to uracil bases. This mechanism has been recently suggested for active demethylation in mouse zygotes (24). A previous study has indicated that the AID/Apobec2/Gadd45/Mbd4 system is not working in zebrafish embryos before zygotic genome activation (33). In our RNA profiles, transcripts for *aid*, *apobec2*, *dctd*, *mbd*, and *gadd45* genes were either absent or present in low amount during early embryogenesis (Table 1). It is unknown whether other deaminases may function together with Unga during early embryogenesis.

Third, Ung may be able to excise oxidation/deamination intermediates of 5-methylcytosines. This kind of activity of Ung was not detected in our *in vitro* glycosylase activity assays. We tried to use embryonic protein extracts from embryos at 4–16-cell stages together with recombinant Unga in these assays, but we still failed to detect removal of 5mC and A, T, or G mispairing with G (data not shown). We suspected that the method we used was not sufficiently sensitive to detect subtle changes in the composition of the substrate DNA. Therefore, more sensitive methods are needed to characterize Ung activity in detail. In addition, identification of oxidation and/or deamination enzymes in early zebrafish embryos will definitely help elucidate mechanisms controlling postfertilization DNA demethylation in this species.

The global DNA methylation level is related to chromatin modifications and genome transcription activity during early embryonic development. Overexpression of *unga* appears to cause an increase of both H3K4me3 and H3K27me3 marks (Fig. 6) as well as an enhancement of transcriptional activity at the pre-MBT stage (256-cell stage) (Fig. 7, *C* and *D*), suggesting that *unga*-mediated global DNA demethylation facilitates chromatin modifications, thereby poising chromatin for transcription activation or repression. Our transcriptome analysis by RNA seq indicated that the majority of affected genes in *unga*-overexpressing embryos were up-regulated and the majority in *unga* knockdown embryos were down-regulated, but still, some genes experienced an opposite change of expression (Fig. 8 and supplemental Tables S2–S5). This phenomenon is understandable given that different modifications of chromatin, availability of transcription factors, and mutual repression of some factors could all contribute to transcription of a specific gene.

A recent paper reports that mouse zygotes depleted of *cytosine deaminase* (*AID*) or *Ung2* have higher levels of 5mC compared with wild-type zygotes (24). It appears that the involvement of the Ung-mediated base excision repair process in active demethylation of zygotes is conserved in vertebrates.

In summary, our findings shed light on the involvement of Ung in postfertilization DNA demethylation and in embryonic survival in zebrafish. Its underlying mechanisms need to be investigated in the future.

## Supplementary Material

Supplemental Data

## Footnotes

2 The abbreviations used are:

5mC: 5-methylcytosine
cMO: control morpholino
DEG: differentially expressed gene
GDM: global DNA methylation
H3: histone 3
hpf: hours postfertilization
MBT: midblastula transition
MO: morpholino
UDG: uracil-DNA glycosylase
Unga: uracil-DNA glycosylase a.
