# Supplementary material for: Uracil-DNA Glycosylase Is Involved in DNA Demethylation and Required for Embryonic Development in the Zebrafish Embryo
Source: J Biol Chem. 2014 Apr 16;289(22):15463–73. doi: 10.1074/jbc.M114.561019 (PMC4140902; doi:10.1074/jbc.M114.561019)
Supplement: Supplemental Data [file supp_289_22_15463__index.html]

Uracil-DNA Glycosylase is involved in DNA demethylation and required for embryonic development in the zebrafish embryo — Uracil-DNA Glycosylase Is Involved in DNA Demethylation and Required for Embryonic Development in the Zebrafish Embryo — Ung in DNA Demethylation and Zygotic Genome Activation — Supplemental Data 

# Uracil-DNA Glycosylase Is Involved in DNA Demethylation and Required for Embryonic Development in the Zebrafish Embryo

## Supplemental Data

**Files in this Data Supplement:**

- Table S1 (.xls, 2.6 MB) - RNA profiles of one-cell and 512-cell embryos.
- Table S2 (.xls, 486 KB) - Up-regulated genes in unga-mcherry overexpressing embryos compared to mcherry-overexpressing embryos at 256-cell stage.
- Table S3 (.xls, 350 KB) - Down-regulated genes in unga-mcherry overexpressing embryos compared to mcherry-overexpressing embryos at 256-cell stage.
- Table S4 (.xls, 381 KB) - Down-regulated genes in unga-MO-injected embryos compared to cMO-injected embryos at 1k-cell stage.
- Table S5 (.xls, 185 KB) - Up-regulated genes in unga-MO-injected embryos compared to cMO-injected embryos at 1k-cell stage.
